# Do foot and mouth disease vaccines affect bovine bull fertility? A systematic review and meta-analysis

**DOI:** 10.1016/j.vas.2026.100677

**Published:** 2026-05-06

**Authors:** Muloongo C Sitali, Limbikani Matumba, Madalitso Chelenga

**Affiliations:** a Department of Biomedical Sciences, School of Veterinary Medicine, The University of Zambia, Lusaka, Zambia; b FoodPlus Research Group, Faculty of Life Sciences, Natural Resources, LUANAR, Lilongwe, Malawi; c Department of Veterinary Clinical Studies, Faculty of Veterinary Medicine, The Lilongwe University of Agriculture and Natural Resources. Lilongwe. Malawi

**Keywords:** Vaccination, Sperm parameters, Bovines, Semen quality, FMD

## Abstract

Foot-and-mouth disease (FMD) is a highly contagious, reportable, and economically devastating disease of cattle and other cloven-hoofed animals. Vaccination against FMD remains the mainstay control strategy in many countries prone to the disease. Evidence shows that vaccinations against FMD could have negative consequences on bull fertility; however, the existence of that threat has received little attention amongst researchers and governments, probably due to weaker evidence from individual reports. This review aimed to systematically gather available evidence from different studies and meta-analyse the data to provide evidence-based pooled estimates of the effects of FMD vaccines on key indicators of bull fertility. To that end, we gathered and analysed published data from PubMed, Web of Science, and Google Scholar to determine the pooled estimates or descriptive synthesis of the effects of FMD vaccine on semen quality, measured before and after administration. A total of 9 articles were included in the meta-analysis, from which semen parameters were pooled using the inverse variance method, while 4 additional studies were synthesized descriptively. The weighted mean difference (WMD) was used to measure the effect sizes, with statistical significance set at *p* < 0.05. Although the FMD vaccination showed no effect on sperm volume between 7–14 days post-vaccination, it significantly increased sperm volume between 28–35 days post-vaccination (WMD 0.04 ml, 95% CI 0.00 to 0.08, *p* = 0.03). While the findings from the narrative synthesis present contrasting outcomes, they collectively suggest that vaccination may influence sperm quality in bovine bulls through physiological pathways potentially involving transient hyperthermia and oxidative stress.

## Highlights

• FMD vaccination may trigger fever and oxidative stress, leading to sperm cell damage, reduced sperm quality, and increased sperm abnormalities in bulls.
• FMD vaccination may transiently influence semen quality in breeding bulls.
• Despite transient changes in sperm parameters, regular vaccination is essential for preventing infectious diseases in breeding bulls.

## 1 Introduction

Foot-and-mouth disease (FMD) is a highly contagious disease of cloven-hoofed animals, including cattle, pigs, sheep, goats, and many wild species (Chimera et al., 2022; Gubbins et al., 2025). In cattle, the characteristic clinical signs include fever, inappetence, lameness, vesicular lesions on the mouth, feet, nares, and teats (Gee et al., 2024). Despite causing relatively lower mortality rates (Gizaw et al., 2024), FMD reduces growth rates, reproductive performance, and milk yield, thereby crippling the overall productivity (Bijari et al., 2025). In FMD-free countries, control has mainly been achieved via depopulation of infected and in-contact animals, combined with restrictions on the movement of animals and their products (Diaz-San Segundo et al., 2017); however, due to the high economic and logistical costs of depopulation, many countries implement emergency vaccination campaigns (Bates et al., 2003; Porphyre et al., 2018). These campaigns often target the entire cattle population within a short period, sometimes coinciding with the breeding season (Garcia-Pintos et al., 2021).

Bull fertility is an important economic driver in cattle production systems, as a single bull can influence the reproductive outcomes of hundreds of females through natural service or artificial insemination (AI) (Sitali et al., 2017; Talluri et al., 2022). Even modest reductions in semen quality or breeding capacity can lead to substantial economic losses through reduced conception rates, prolonged calving intervals, and increased culling or replacement costs (Kastelic, 2013). Despite this importance, reproductive monitoring of bulls is rarely incorporated into national or institutional policy frameworks, including vaccine safety programs, where post‑vaccination fertility assessments are not routinely mandated. This policy gap is compounded by a scientific gap, as few large-scale or systematic studies have evaluated the effects of vaccines or other interventions on bull fertility. These deficiencies highlight the need for comprehensive evaluations that synthesize available evidence and clarify whether FMD vaccination poses measurable reproductive risks to bulls.

Bull fertility depends on the production of high-quality sperm (Anwar et al., 2024; Sitali et al., 2016), adequate physical capacity for breeding, and sufficient libido and mating ability necessary for successful insemination (Hoflack et al., 2006). Semen quality is commonly determined by measuring important parameters such as volume, motility, concentration, morphology, and viability (Yoon et al., 2022). If vaccinations affect bull fertility, one plausible mechanism is oxidative stress (OS) resulting from excessive production of reactive oxygen species (ROS) (Gupta et al., 2025). The sperm plasma membrane is rich in polyunsaturated fatty acids, making spermatozoa particularly susceptible to ROS-induced damage, which can impair motility, membrane integrity, acrosomal function, and DNA integrity (Angrimani et al., 2017; Perumal et al., 2013b).

Beyond the aforementioned, the vaccine-induced hyperthermia can also affect semen quality. Spermatogenesis normally occurs at approximately 34–35 °C, which is several degrees below core body temperature (Jorban et al., 2024). Accordingly, hyperthermia is known to cause male infertility by impairing the entire spermatogenesis process, including damage to the spermatogonial stem cells, sertoli cells, and fully formed spermatozoa (Jorban et al., 2024). Previous studies have shown that heat stress reduces daily sperm production, decreases motility, increases morphological abnormalities, and may induce sperm DNA fragmentation and apoptosis through OS mediated pathways across species (Burgio et al., 2024; Jorban et al., 2024; Kastelic et al., 2019; Rao et al., 2016,; 2015; Rockett et al., 2001; Ziaeipour et al., 2021). Despite these potential mechanisms, the reproductive effects of FMD vaccination on bulls remain poorly understood.

To address the gap, we systematically gathered available data from published studies and conducted a meta-analysis to estimate the effect of FMD vaccines on key semen quality parameters. This systematic review and meta-analysis aimed to provide evidence to address the following question: Does vaccination against FMD result in measurable changes in semen quality parameters such as sperm concentration, motility, viability, and volume in bulls*?*. By consolidating the current body of evidence, this study identifies knowledge gaps and research priorities related to vaccine-induced fertility effects in bulls and seeks to inform an integrated FMD management approach and policy directions that will promote FMD control while upholding fertility sustainability in bovines.

## 2 Methods

### 2.1 Eligibility criteria

#### 2.1.1 Inclusion criteria

The inclusion criteria followed the PICOS (Population, Intervention, Comparison, Outcome, and Study design) framework to identify the eligible studies. The included studies were those that involved experiments using FMD vaccines in bovines published before November 2025. The PICOS was defined as follows: (1) Population – bovine bulls in healthy condition without disease that could harm their sperm quality (2) Intervention – FMD vaccinations, (3) Comparison – semen parameters before and after vaccinations of the experimental animals or unvaccinated controls (4) Outcome- effect of vaccination on sperm parameters such as sperm motility, sperm concentration, sperm morphology etc. (5) Study design – experimental studies where relevant outcomes were compared between post and pre-vaccination states, or with unvaccinated controls.

#### 2.1.2 Exclusion criteria

Studies were excluded from this review if they focused on outcomes other than the effects of FMD vaccination on semen quality in bovine bulls. Reviews, letters to the editor, comments, conference proceedings, encyclopedias, and studies published after November 2025 were also excluded.

### 2.2 Information sources

Databases from which information was obtained included the Web of Science, PubMed, and Google Scholar.

### 2.3 Search strategy

The first online search in the three databases was conducted on 3rd January 2025 using one search phrase. An additional search was conducted on 9th December 2025 to identify any newly published articles. A thorough literature search focused on the effects of vaccination on sperm quality in bovine bulls. The online database search was conducted using the search phrase, which read: (Foot and Mouth Disease OR FMD) AND (vaccination OR vaccine) AND (bovine OR cattle OR buffalo OR bull) AND (Semen OR Sperm OR spermatozoa OR “sperm quality” OR “sperm parameters” OR “seminal attributes” OR “seminal traits”).

### 2.4 Study selection

The study selection process followed the Preferred Reporting Items for Systematic Reviews and Meta-Analyses (PRISMA) guidelines (Moher et al., 2015) (Supplementary Table S1). Two reviewers independently evaluated the titles and abstracts of all publications found. In the event of a dispute, a consensus was reached through discussion with the third author regarding which articles should be selected for full-text review. The two reviewers then independently evaluated the full texts to assess the appropriateness of the articles for inclusion. A descriptive synthesis for studies that could not be incorporated into the meta-analysis was done. The protocol for this systematic review and meta-analysis was not registered in PROSPERO due to timing constraints, as data extraction had already commenced before the initiation of the registration process. While this departure from best practice is acknowledged, all efforts were made to ensure methodological transparency and rigor throughout the review process.

### 2.5 Data extraction process

Two reviewers conducted data extraction independently. Data extracted included: the title of the article, authors, year of publication, objective, breed of the bulls used, age of the bulls, semen collection method, sperm assessment time points post-vaccination, and the means, sample size, and standard errors for the effect of vaccination on sperm quality parameters.

### 2.6 Statistical analysis

Statistical analysis was performed using the IBM Statistical Package for Social Sciences Version 31. The standard deviations (SD) were calculated using the Revman online calculator for SD calculation from the formula SD = SE x √N (where SE is the standard error, and N is the sample size). As a first step, potential publication bias was assessed through visual inspection of funnel plot asymmetry, following the recommendations of Sterne and Egger (2001), and further evaluated using Egger’s regression-based test. Heterogeneity was identified using the inconsistency (I^2^) metric. Levels of statistical heterogeneity were classified into three categories; I^2^ < 30% was considered to be low, I^2^ of 30–50% as moderate, and I^2^ > 50% as high (Xu et al., 2023). Semen quality parameters from bovine bulls were pooled using the inverse variance method with a fixed effect model when I^2^ was <50%. Otherwise, a random effects model was used for analysis (I^2^ ≥ 50%), which denotes a statistical heterogeneity among the studies (Hooijmans et al., 2014a; Xu et al., 2023). The weighted mean difference (WMD) was used as an effect summary measure, and statistical significance was set to *p* < 0.05.

## 3 Results

### 3.1 Selection of eligible studies

**Fig. 1:**
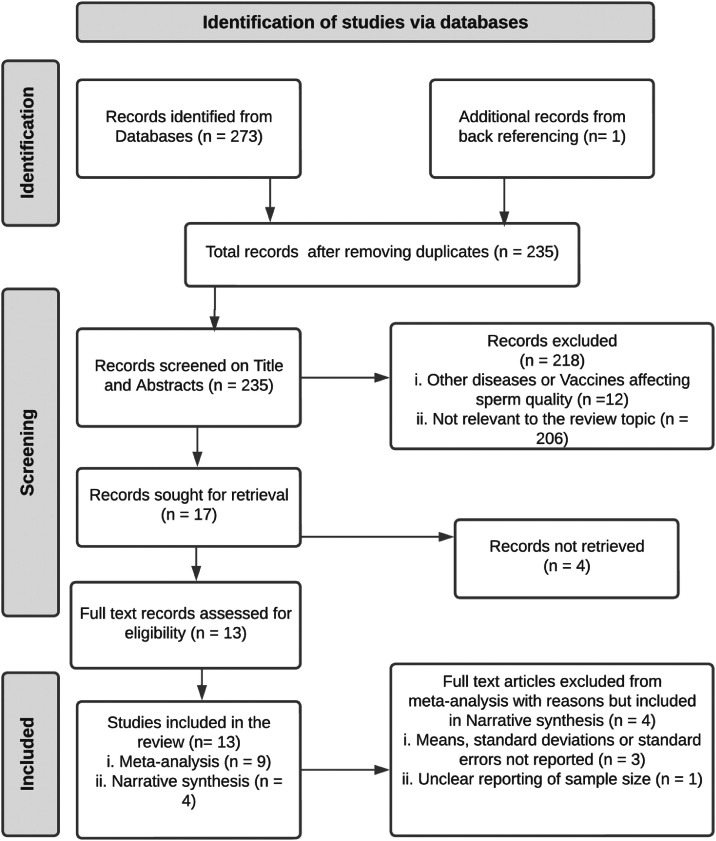
PRISMA flow diagram.

A total of 274 articles were found in the databases PubMed, Web of Science, and Google Scholar (Fig. 1). After removing duplicates, we reviewed 235 papers, of which only 13 studies met the inclusion criteria. Nine (9) articles were included in the meta-analysis, while four (4) studies formed the basis of the narrative synthesis. The reasons for excluding the 4 articles from the meta-analysis are shown in Fig.1.

### 3.2 Risk of bias assessment

**Fig. 2:**
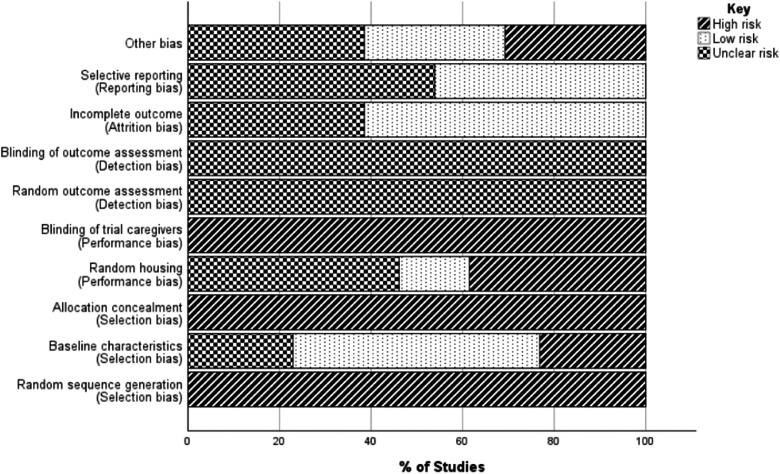
Risk of bias in included studies using the SYRCLEs risk of bias tool.

All articles included in this study were assessed for risk of bias using the Systematic Review Centre for Laboratory Animal Experimentation (SYRCLES) risk of bias tool for animal studies (Hooijmans et al., 2014b). Two independent reviewers conducted the risk of bias assessment of the included studies. The results of the risk of bias assessment are presented in Fig. 2.

### 3.3 Quality assessment of the studies included

**Fig. 3:**
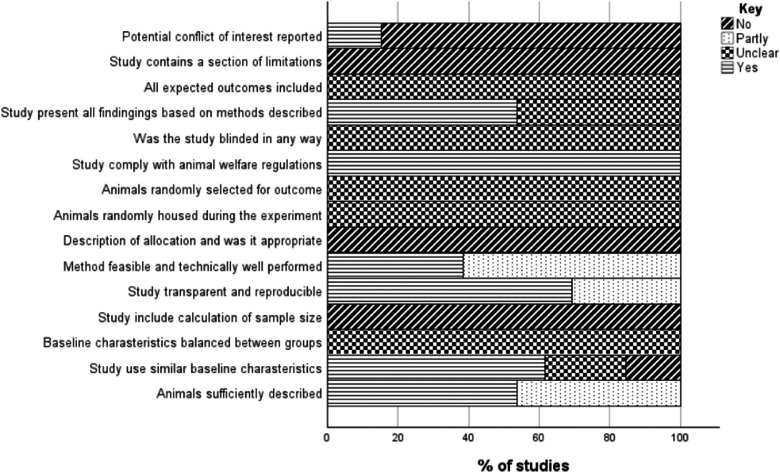
Quality assessment in the Included studies.

The quality of the articles included in the review was evaluated by Muloongo Chinani Sitali (MCS) and Madalitso Chelenga (MC) using the Critical Appraisal of Methodological (technical) Quality, Quality of Reporting and Risk of Bias in Animal Research (CRIME-Q) tool (Andersen et al., 2025) (Fig. 3).

### 3.4 Characteristics of the studies included in the narrative synthesis

Table 1 summarizes the characteristics of the studies included in the descriptive synthesis. Four studies were included in the narrative synthesis, all of which were conducted in India, with no eligible studies reported from other FMD-endemic regions. The studies evaluated different bovine breeds, including Holstein Friesian, Mithun, and Surti bulls, with ages ranging from 2 to 7 years. Two studies used trivalent vaccines and two used polyvalent vaccines. In all four studies, the reproductive effect of FMD vaccination was assessed using pre- and post-vaccination comparisons. Sperm parameters were monitored for up to 84 days post-vaccination.

**Table 1:** Description of the studies included in a narrative synthesis.

Country	Breed	Age	Type of FMD vaccine	Control	Outcome measures	Analysis duration PV (days)	Reference
India	Mithun	4–6	Trivalent (Raksha-Ovac)	BV	V, C, MA IM, CN, %LS, AI, PMI	84	(Perumal et al., 2013a)
India	HF	2–7	Trivalent (Raksha Ovac)	BV		30	(Saha et al., 2011)
India	HF	2.5–3	Polyvalent (Intervet)	BV	V, CN, IM, %LS, SA	75	(Kumar et al., 2007)
India	Surti	3.5–4	Polyvalent (IVRI Bangalore)	BV	V, C, PM	84	(Kammar & Gangadhar, 1998)

HF: Holstein Friesian, BV: Before vaccination, MA: Mass activity, IM: Initial motility, V: volume, C: Color, CN: Concentration, %LS: % Live sperm, AI: Acrosome integrity, PMI: Plasma membrane integrity, SA: Sperm abnormalities, PM: Progressive motility.

### 3.5 Characteristics of the studies included in the meta-analysis

Nine eligible studies were included in the meta-analysis, and their characteristics are summarised in Table 2. Of the nine studies, one was conducted in Indonesia and one in Iran, with the remaining seven conducted in India. Across studies, semen was collected from bovine bulls aged 3–12 years using either an artificial vagina or rectal massage; however, five studies did not report the age of the animals at the time of semen collection. Four studies evaluated trivalent vaccines, two used tetravalent vaccines, one study used a polyvalent vaccine, and one assessed the 6PD50 Aftopor vaccine by comparing semen parameters before and after vaccination. One study did not report the type of vaccine used. Only one study included unvaccinated controls, whereas the other eight studies compared pre-vaccination sperm parameters to post-vaccination sperm parameters. Due to inconsistencies in reporting and limited availability of data on other semen parameters, only concentration, volume, and viability were included in the meta-analysis. Additionally, some studies could not be incorporated in subgroup analyses because post-vaccination assessment time points for semen parameters were not reported.

**Table 2:** Description of the studies included in the meta-analysis.

Country	Breed	Age (Yr)	Vaccine type	Control	Sperm parameters meta-analysed	Analysis duration after vaccination (days)	Reference
India	Vrindavani	NR	Trivalent(serotypes O, A, Asia 1)	BV	CN, V, VB	7–42	(Bhardwaj et al., 2023)
Indonesia	Bali	6–12	6PD50 Aftopor (serotypes 0–3039, O-MANISA, A22 IRAQ)	BV	CN, V	21	(Prihatin et al., 2023)
Iran	HF	3–5	Polyvalent (serotypes 02,016, A13, A15, Asia 1)	BV	CN, V	15–30	(Forough et al., 2022)
India	HF	3–5	Trivalent (serotypes O, A, Asia 1) Rakshac-Ovac	BV	CN, V, VB	15–30	(Das Gupta et al., 2018)
India	HF × Sahiwal	NR	Trivalent (serotypes O, A, Asia 1)	BV	CN, V	28–150	(Sirohi et al., 2016)
India	Mithun	4–6	Trivalent (serotypes O, A, Asia 1)	BV	CN	84	(Perumal et al., 2013b)
India	Karan fries and Murrah buffalo	NR	Tetravalent (serotypes O, A, C, and Asia 1 strains)	NV	CN, V	NR	(Bhakat et al., 2010)
India	Sahiwal	NR	Tetravalent (serotypes O, A, C, and Asia 1 strains)	BV	CN, V	NR	(Bhakat et al., 2008)
India	HF, Jersey, HF × Jersey × Zebu	NR	NR	BV	V	7	(Mangurkar et al., 2000)

NR: Not reported, HF: Holstein Friesian, BV: Before vaccination, NV: Non- vaccinated, V: Volume: CN: Concentration, VB: Viability.

### 3.6 Effect of vaccination on sperm parameters: evidence from a descriptive synthesis

The impact of FMD vaccination on semen parameters in the studies included in the narrative synthesis is summarized in Table 3. Three studies reported the effect of the FMD vaccine on the total defects (Kumar et al., 2007; Perumal et al., 2013a; Saha et al., 2011). Two of these reported a higher proportion of total defects post-vaccination compared to pre-vaccination levels; however, the pre-vaccination proportion of tail, mid-piece, and head defects did not differ from the post-vaccination levels (Saha et al., 2011). Two studies reported a post-vaccination decrease in semen volume based on data from ejaculates from eight Mithun bulls and twenty-five Holstein Friesian (HF) bulls (Perumal et al., 2013a; Saha et al., 2011), whereas one study observed an increase in semen volume after vaccination in ejaculates from four HF bulls (Kumar et al., 2007).

**Table 3:** FMD vaccination effects on sperm parameters for the studies included in the narrative synthesis.

Mass Activity	Initial Motility	Individual Motility	% Live sperm	Concentration	Volume	Head defect	Midpiece defect	Tail defect	Total defect	Acrosome Integrity	Plasma Membrane Integrity	Progressive Motility	Study
D	NA	D	D	D	D	NA	NA	NA	I	D	D	NA	(Perumal et al., 2013a)
NA	D	NA	D	NE	D	NE	NE	NE	NE	NA	NA	NA	(Saha et al., 2011)
NA	D	NA	D	D	I	NA	NA	NA	I	NA	NA	NA	(Kumar et al., 2007)
NA	NA	NA	NA	NE	NE	NA	NA	NA	NA	NA	NA	D	(Kammar & Gangadhar, 1998)

Key: D: Decreased, I: Increased, NA: Not Assessed, NE: No Effect.

Two studies reported reduced initial motility after vaccination in ejaculates from 25 to 4 HF bulls (Kumar et al., 2007; Saha et al., 2011). Furthermore, two studies reported decreases in sperm concentration following vaccination, based on ejaculates from eight Mithun bulls and four HF bulls (Kumar et al., 2007; Perumal et al., 2013a). In contrast, two other studies found no significant post-vaccination changes in sperm concentration (Kammar & Gangadhar, 1998; Saha et al., 2011). Three studies (Kammar & Gangadhar, 1998; Kumar et al., 2007; Perumal et al., 2013a) did not evaluate the effects of vaccination on the head, midpiece, and tail defects.

In addition, three studies reported post-vaccination decreases in the percentage of live sperm in ejaculates from eight Mithun bulls, and from twenty-five and four HF bulls (Kumar et al., 2007; Perumal et al., 2013a; Saha et al., 2011).

### 3.7 Effects of FMD vaccine on sperm parameters meta-analysis

#### 3.7.1 Effect of FMD vaccine on sperm concentration, viability, and volume

**Fig. 4:**
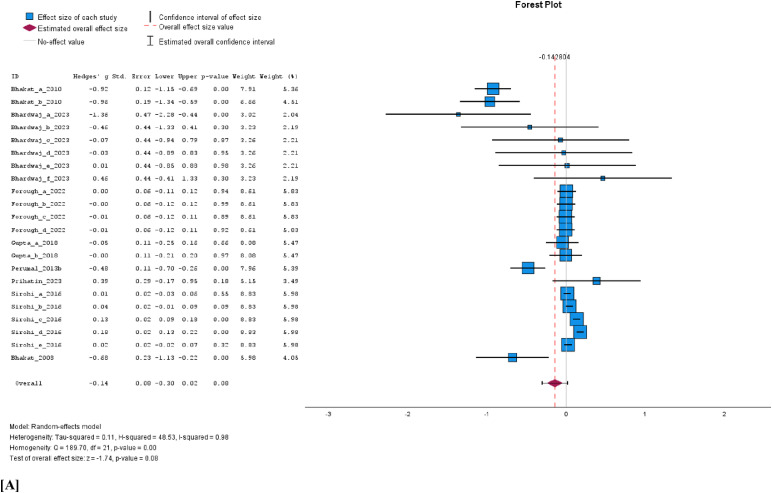

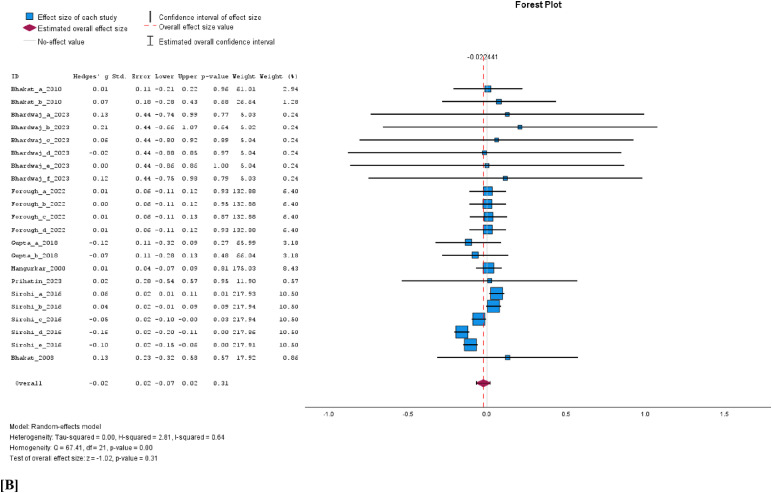

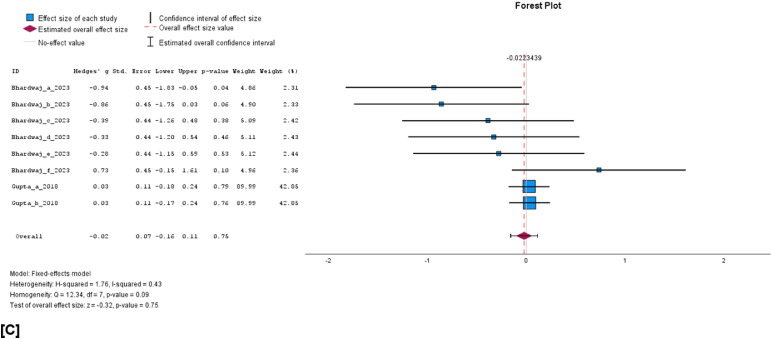
Forest plots of cumulative sperm parameters before and after vaccination; [A] concentration (x10^6^/ml), [B] Volume (ml), and, [C] % viability.

**Fig. 5:**
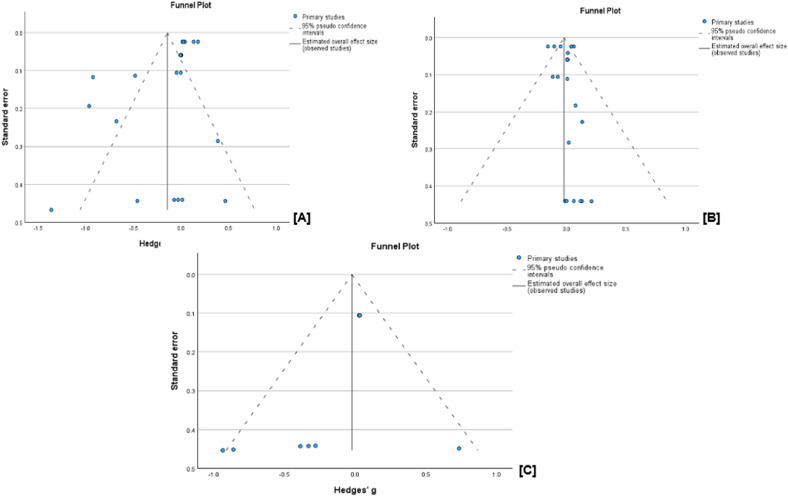
Funnel plots of cumulative sperm parameters before and after vaccination; [A] concentration, [B] volume, and [C] viability.

The cumulative analysis of the vaccination effect on sperm concentration, viability, and volume after vaccination showed that none of the semen parameters was affected by FMD vaccination. In a comparison of vaccinated versus unvaccinated group, the WMD for sperm concentration was −0.14 × 10^6^/ml (95% C1, −0.30 to 0.02, *p* = 0.08; Fig. 4A), volume −0.02 ml (95% CI, −0.07 to 0.02, *p* = 0.31; Fig. 4B) and viability −0.02% (95% CI, −0.16 to 0.11, *p* = 0.75; Fig. 4C). The results of a random effects model indicated high heterogeneity among the studies for concentration (I^2^ = 98%, *p* < 0.001) and volume I^2^ = 64%, *p* < 0.001), while viability showed a non-significant moderate heterogeneity (I^2^ = 43%, *p* = 0.09). Evidence of significant publication bias for sperm concentration, viability, and volume can be ignored as the Eggers regression-based test showed no evidence of publication bias (*p* > 0.05). Besides, the studies appear to be evenly scattered on the funnel plots (Fig. 5A, 5B, and 5C).

#### 3.7.2 Subgroup analysis of sperm concentration, viability, and volume

Given the substantial heterogeneity observed, subgroup analyses were performed based on specific post-vaccination time points for semen parameter assessments.

##### 3.7.2.1 Effect of FMD vaccine on sperm concentration: evidence from a meta-analysis

**Fig. 6:**
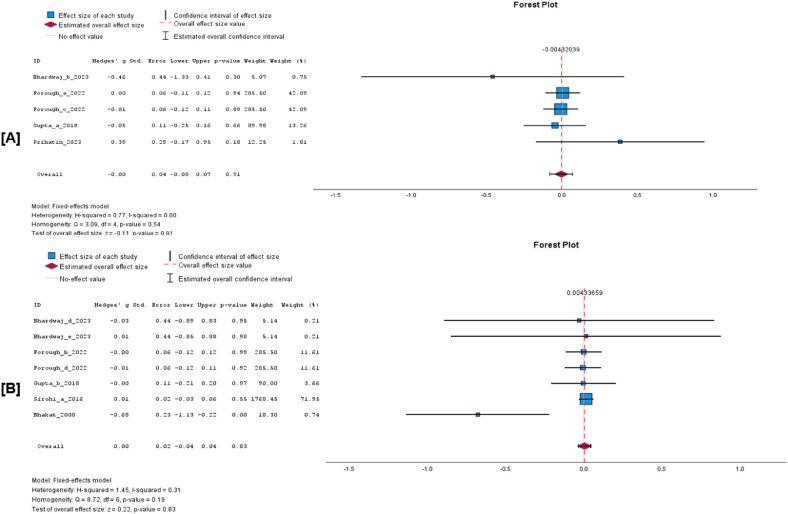
Forest plots of sperm concentration (x10^6^/ml) before and after FMD vaccination. Forest plot [A] Concentration at 14 days PV, [B] Concentration at 28 to 35 days PV.

Evidence from the meta-analysis revealed no significant difference in sperm concentration at 14 days after vaccination (Fig. 6A; WMD 0.00 × 10^6^/ml, 95% CI −0.08 to 0.07, *p* = 0.91; I^2^ = 0%, *p* = 0.54). Similarly, there was no significant difference in sperm concentration between the vaccinated and unvaccinated bull at 28 to 35 days post-vaccination (Fig. 6B; WMD 0.00 × 10^6^/ml, 95% CI −0.04 to 0.04, *p* = 0.83; I^2^ = 31%, *p* = 0.19).

##### 3.7.2.2 Effect of FMD vaccine on sperm volume: evidence from a meta-analysis

**Fig. 7:**
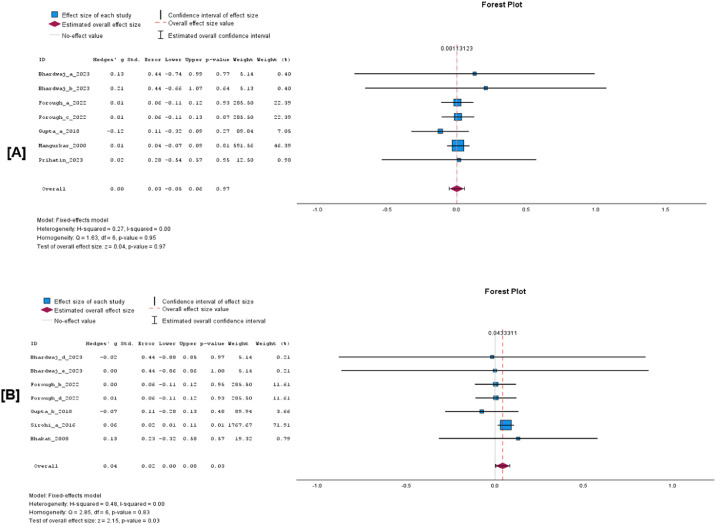
Forest Plots of sperm volume (ml) before and after FMD vaccination. Forest plot [A] volume at 7 to 14 days PV, [B] volume at 28 to 35 days PV.

There was a significant difference in sperm volume 28 to 35 days post-FMD vaccination (Fig. 7B; WMD 0.04 ml, 95% CI 0.00 to 0.08, *p* = 0.03; I^2^ = 0%, *p* = 0.83). However, there was no significant difference in sperm volume between 7 and 14 days following vaccination (Fig. 7A; WMD 0.00 ml, 95% CI −0.05 to 0.06, *p* = 0.97; I^2^ = 0%, *p* = 0.95).

##### 3.7.2.3 Effect of FMD vaccination on sperm viability: evidence from a meta-analysis

No significant changes in sperm viability were observed at either 14 to 21 days or 28 to 35 days post-vaccination (Supplementary Fig. 1 and Supplementary Fig. 2).

## 4 Discussion

This systematic review and meta-analysis provide evidence that vaccination against endemic bovine FMD, can induce measurable alterations in semen quality among bulls. Despite some variability across studies, reductions in sperm concentration and motility along with an increase in total sperm defects were observed. While semen volume showed variability across studies, meta-analysis indicated a transient increase in semen volume approximately four weeks post-vaccination. These recurrent trends support the hypothesis that vaccination may transiently affect testicular or epididymal function. Although temporary, such effects may be consequential in systems relying on tight breeding schedules or artificial insemination (AI). These findings also highlight the importance of evaluating reproductive outcomes in the context of newer FMD vaccine technologies, including recombinant capsid protein vaccines (Wang et al., 2023; Yu et al., 2025), adenoviral-vectored vaccines (Schutta et al., 2016; Sitt et al., 2019), and nanoparticle-based formulations (Cao et al., 2025; Lu et al., 2024). Comparative studies showed no significant effect of Peste des petits ruminants and bluetongue vaccination on sperm quality in bucks and rams, respectively (Leemans et al., 2012; Raji et al., 2024). Conversely, one boar treated with an oil-based vaccine against porcine circovirus type-2 showed a temporarily impaired semen quality after elevated body temperature (Caspari et al., 2014). This similar vaccine-associated fertility change, further support the biological plausibility of such effects across mammalian species.

One key biological mechanism contributing to altered semen quality is oxidative stress (OS). Two studies reported increases in total sperm abnormalities after vaccination, although they did not detail the proportions of head, midpiece, or tail defects. Notably, elevated reactive oxygen species (ROS) following vaccination can impair sperm motility, morphology, and DNA integrity (Awda et al., 2009; Bollwein & Bittner, 2018; Khoi et al., 2021). ROS elevation may also cause membrane lipid peroxidation and chromatin fragmentation (Kumar et al., 2002; Wu et al., 2020). Supplementation with antioxidants such as ergothioneine, vitamins C and E, and selenium has shown promise in mitigating OS effects (Fadl et al., 2024; Younus et al., 2024). Future research would benefit from incorporating advanced fertility assessment tools including; sperm DNA fragmentation, ROS quantification, and computer-assisted sperm analysis (CASA), which remain underutilized in current FMD vaccine research. Besides, further investigation is warranted to assess whether these improvements translate into enhanced reproductive outcomes in vivo*.*

Another important mechanism involves febrile responses post-vaccination. Previous studies have reported transient elevations in body temperature following vaccination against both lumpy skin disease (LSD) and FMD, supporting the evidence that short-lived systemic reactions are a common post-vaccination response (Ferreira et al., 2016; Haegeman et al., 2023) that may disrupt testicular thermoregulation and spermatogenesis. Even short-lived hyperthermia can affect meiotic division, mitochondrial function, and chromatin condensation (Gupta et al., 2025; Ribeiro et al., 2025). These disruptions may persist throughout the 61-day spermatogenic cycle. In one LSD vaccination study, body temperatures peaked at 40.6 °C but normalized within a week (Haegeman et al., 2023), although semen recovery timelines were not reported. Acute immune responses and hypersensitivity reactions may further exacerbate testicular or epididymal damage, potentially reducing sperm viability.

In addition to the scientific implications, these findings have notable translational relevance. For breeding and AI centers, even temporary declines in semen quality may reduce conception rates and compromise genetic improvement objectives. Economically, disruptions to semen production or bull fertility can lead to missed breeding windows and financial losses. Moreover, recognizing transient fertility changes as part of a broader immune-metabolic interaction reinforces the need to consider animal health, productivity, and welfare as interconnected domains. Monitoring semen quality in national surveillance programs for bull health could also offer early warning indicators for transient fertility impacts associated with vaccination against FMD. The underlying physiological responses and their mitigation could also inform fertility-preserving vaccine design across other livestock species with similar thermoregulatory and spermatogenic profiles. The integration of fertility endpoints into national and global vaccine safety frameworks may be warranted. This approach aligns with increasing international attention to comprehensive vaccine safety and is consistent with the standards and guidance of the World Organisation for Animal Health (WOAH, 2022).

The transient increase in semen volume observed approximately four weeks after vaccination aligns with the expected physiological adjustments that accompany immune activation and mild febrile responses. These short-lived changes likely reflect temporary disruptions in thermoregulation, epididymal function, or accessory gland secretions rather than lasting impairment of spermatogenesis. Similar temporal patterns have been reported in other livestock species following vaccination or febrile stress, which supports the biological plausibility of these effects. The subsequent recovery toward baseline levels further indicates that the changes are reversible and adaptive. However, the current evidence base remains limited because most studies were small and geographically concentrated, which restricts the ability to generalize the findings. Future research should therefore use standardized semen evaluation protocols, include a wider range of breeds and production systems, and incorporate longer and more consistent follow-up periods.

Although very few studies have directly evaluated the impact of FMD on semen quality, the limited evidence available (e.g. AL-Badry et al., 2012), together with findings from other viral infections such as bluetongue virus (Martinelle et al., 2025; Muller et al., 2010) and Bovine viral diarrhoea virus (BVDV) (El-mohamady et al., 2020), indicates that natural viral disease can cause substantial reproductive impairment in males. In contrast, this review demonstrates that vaccine-associated alterations in semen quality are generally mild, transient, and primarily attributable to short-lived febrile or OS responses rather than any lasting testicular damage. Accordingly, vaccination remains clearly protective when weighed against the markedly greater fertility risks posed by viral diseases. From a practical standpoint, FMD vaccination in semen producing bulls should ideally be scheduled outside intensive semen collection periods or breeding season, with cryopreservation undertaken after the brief post-vaccination febrile window. In cases where clinically significant fever develops, timely veterinary-supervised anti-inflammatory therapy may help mitigate thermal stress and maintain optimal semen quality.

The present study is subject to several limitations. Most of the included studies were conducted in India, with limited regional diversity. While the physiological mechanisms involved are broadly conserved, this geographic concentration may limit the ability to account for regional variations in breed susceptibility, vaccine formulations, and management practices. Heterogenous study designs, relatively small sample sizes, and incomplete reporting constrained the robustness of the meta-analysis conclusions. Besides, variability among studies may arise from differences in bull breed, vaccination protocols, management systems, semen collection intervals, and the analytical methods used to evaluate semen quality. The number of studies per semen parameter subgroup was insufficient to fully characterize the range and duration of vaccine-induced effects, and many failed to assess the post-vaccination recovery timeline. Semen evaluation protocols varied widely across studies, including the parameters assessed and the timing of post-vaccination measurement.

Important indicators such as midpiece and tail defects or DNA fragmentation were frequently omitted or inconsistently reported. Moreover, critical biological modifiers such as bull age, breed, immune status, nutritional condition, vaccine type and management practices were seldom analysed or stratified. This lack of standardisation impedes comparability across studies and undermines generalizability. Furthermore, due to the relatively small number of included studies, the assessment of publication bias using funnel plots and Egger’s regression test is inherently limited. This also prevented a detailed categorization of results according to vaccine formulation, which may induce varying levels of systemic inflammation. Moreover, many of the studies included in this meta-analysis relied on pre- and post-vaccination comparisons rather than vaccinated versus unvaccinated control group designs. Although pre- and post-vaccination designs provide useful insights into temporal changes following vaccination, pooling results from studies with different methodological approaches may introduce potential bias, as changes in semen parameters could also be influenced by time-dependent factors unrelated to vaccination. Future studies employing well-controlled experimental designs, including appropriate unvaccinated control groups, would help strengthen causal inference and improve the robustness of evidence in this area. Another limitation of this study is that the systematic review protocol was not prospectively registered in PROSPERO; however, the review was conducted in accordance with the PRISMA guidelines, and the search strategy, inclusion criteria, and analytical methods were predefined to maintain transparency and methodological consistency. Despite these limitations, this synthesis highlights critical research gaps and provides actionable insights for veterinarians and livestock managers.

## 5 Conclusion and future perspectives

This systematic review and meta-analysis reveal that vaccination against endemic FMD is associated with transient changes in semen quality, particularly affecting sperm concentration, percentage of live sperm, motility, and volume. These alterations are mediated through physiological mechanisms, including OS and febrile responses, with implications for testicular and epididymal function. Although reversible in most cases, these effects can be consequential when they coincide with breeding periods, AI programs, or semen collection windows. In considering future directions, it is important to recognize ongoing historical developments in vaccine technology, including the shift from conventional live or inactivated platforms to recombinant subunit, peptide-based, and emerging mRNA vaccines, which may mitigate febrile and OS responses that influence fertility outcomes.

To strengthen the reliability and impact of future research, investigators should adopt standardised semen evaluation protocols, enroll diverse bull populations across endemic and non-endemic regions, and control for key biological variables such as age, breed, immune status, and nutritional condition. Additionally, future studies should explore emerging areas such as genetic selection and breed related differences in reproductive resilience to vaccination. Studies should incorporate both molecular and functional endpoints, such as ROS levels, DNA integrity, fertilisation rates, embryo development, and live birth outcomes. Investigating antioxidant supplementation strategies, tracking stress hormone biomarkers, and clearly defining recovery timelines are also priorities. Precision reproductive monitoring technologies, including wearable biosensors and artificial intelligence-assisted semen analysis, represent a key area of progress that may enable earlier detection and more accurate characterisation of vaccine-associated reproductive effects. Importantly, fertility studies using in vivo and in vitro systems will be key to translating semen changes into reproductive impact.

From a policy and application perspective, understanding vaccination timing with reproductive management plans is essential whenever feasible. High-value bulls in AI programs may benefit from fertility monitoring or targeted antioxidant support. Additionally, reproductive outcomes should be integrated into national vaccine safety monitoring systems, aligning with broader trends among regulatory bodies such as the WOAH to incorporate fertility endpoints in vaccine safety evaluations. Addressing vaccine hesitancy through education and transparent communication remains essential. Outreach programs should emphasize that while some reproductive effects may occur, they are typically short-lived and manageable. Collaborative engagement among veterinarians, reproductive physiologists, immunologists, and livestock extension specialists will be essential to designing vaccination strategies that simultaneously promote disease control and safeguard reproductive efficiency in bovines.

## Consent for publication

All the authors have read and approved the final manuscript.

## Availability of data and material

All relevant data are within the article and its supporting documents.

## Funding

The Authors received no specific funding for this work.

## Ethics statement and consent to participate

Not applicable.

## CRediT authorship contribution statement

**Muloongo C Sitali:** Writing – review & editing, Writing – original draft, Visualization, Validation, Methodology, Investigation, Formal analysis, Data curation, Conceptualization. **Limbikani Matumba:** Writing – review & editing, Methodology. **Madalitso Chelenga:** Writing – review & editing, Methodology, Investigation.

## Declaration of competing interest

The authors declare that they have no known competing financial interests or personal relationships that could have appeared to influence the work reported in this paper.

## Appendix Supplementary materials

Image, application 1

Image, application 2

Image, application 3
